# Potential blood-based markers of celiac disease

**DOI:** 10.1186/1471-230X-14-176

**Published:** 2014-10-09

**Authors:** Hanna Bragde, Ulf Jansson, Mats Fredrikson, Ewa Grodzinsky, Jan Söderman

**Affiliations:** Division of Medical Diagnostics, Ryhov County Hospital, Jönköping, Sweden; Department of Pediatrics, Ryhov County Hospital, Jönköping, Sweden; Department of Clinical and Experimental Medicine, Division of Occupational and Environmental Medicine and Linköping Academic Research Center (LARC), Linköping University, Linköping, Sweden; Östergötland County Council & Department of Health and Medicine, Division of Pharmacological Research, R&D Unit in Local Health Care, Linköping University, Linköping, Sweden

**Keywords:** Celiac disease, Molecular diagnostics, Blood-based biological markers

## Abstract

**Background:**

Blood-based diagnostics has the potential to simplify the process of diagnosing celiac disease (CD). Although high levels of autoantibodies against tissue transglutaminase (anti-TG2) are strongly indicative of active CD, several other scenarios involve a need for additional blood-based CD markers.

**Methods:**

We investigated the levels of messenger RNA (mRNA) in whole blood (n = 49) and protein in plasma (n = 22) from cases with active CD (n = 20), with confirmed CD and normalized histology (n = 15), and without a CD diagnosis (n = 14). Group differences were analyzed using Kruskal-Wallis one-way analysis of variance by ranks. We also investigated correlations between levels of potential markers, histopathology according to the modified Marsh scale, and CD risk gradient based on HLA type, using Spearman rank correlation. The relation between HLA-DQ2 gene dose effect and the expression levels of selected blood-based markers was investigated using the Mann–Whitney U test. Finally, the diagnostic performance of anti-TG2, potential blood-based CD markers, and logistic regression models of combined markers was evaluated using receiver operating characteristic (ROC) curve analysis.

**Results:**

*CXCL11* protein levels and *TNFRSF9* and *TNFSF13B* mRNA levels were identified as potential CD markers. These are all affected by or involved in the regulation of the NF-κB complex. *CXCL11* protein levels and *IL21* and *IL15* mRNA levels were correlated with histopathology according to the modified Marsh scale, as were the established CD markers. HLA genotype risk and HLA-DQ2 gene dose effect did not show any significant relations with either the potential CD markers or the established CD markers. ROC curve analysis revealed a slight, non-significant increase in the area under the curve for the combined use of anti-TG2 and different constellations of potential blood-based CD markers compared to anti-TG2 alone.

**Conclusions:**

The CD markers identified in this study further emphasize the significance of components related to NF-κB regulation in relation to CD. However, the relevance of *CXCL11*, *TNFSF13B*, *TNFRSF9*, and other NF-κB interacting proteins recognized by pathway analysis, needs to be further investigated in relation to diagnosis and monitoring of CD.

**Electronic supplementary material:**

The online version of this article (doi:10.1186/1471-230X-14-176) contains supplementary material, which is available to authorized users.

## Background

Celiac disease (CD) is defined as a “chronic small intestinal immune-mediated enteropathy precipitated by exposure to dietary gluten in genetically predisposed individuals” [1]. Autoantibodies against tissue transglutaminase (TG2) can be found in the blood, as can antibodies against endomysium, deamidated gliadin (DGP), and native gliadin (GL); and infiltration of intraepithelial lymphocytes (IELs) in the epithelium, elongation of the crypts, and destruction of villi is seen in the small intestine [2]. A strong genetic component is evident in CD, with the strongest association found in the *HLA* region [3, 4], primarily with DQ2 (DQA1*05/DQB1*02) and in a minority of CD patients with DQ8 (DQA1*0301/DQB1*0302) [5, 6]. There is a gene dose effect of HLA-DQ2 [7], and risk gradients based on HLA type have been calculated [8, 9]. A number of other risk loci containing multiple candidate genes have been associated with CD [3, 4, 10–12].

Current CD diagnostics primarily include antibody detection, mainly of Immunoglobulin A (IgA) autoantibodies against TG2 (anti-TG2), and confirmation of the diagnosis by histopathologic assessment of small intestinal biopsies [13]. The European Society of Paediatric Gastroenterology, Hepatology and Nutrition (ESPGHAN) Working Group on Coeliac Disease Diagnosis suggested in 2012 that children and adolescents with clear symptoms and anti-TG2 levels over 10 times the upper limit of normal (ULN), with a remission of symptoms on a gluten-free diet (GFD), could be diagnosed with CD without histopathologic assessment of an intestinal biopsy [14]. Additionally, the presence of HLA-DQ2 or DQ8 should be verified.

We have previously developed a discriminant analysis model based on gene expression data in duodenal biopsies. This model can discriminate between biopsies with and without histopathologic alterations indicative of CD, and also indicate the level of histologic damage as well as mucosal recovery on a GFD [15]. The identification of blood-based markers which could reinforce the diagnostic value of anti-TG2, and perhaps indicate the level of histologic damage and mucosal recovery on GFD, would further simplify CD diagnostics.

In the present study, we investigated levels of candidate CD markers (messenger RNA [mRNA] and protein) in blood from celiac and non-celiac cases, and considered the possible added value of these candidate markers. We also explored the relations between candidate markers and enteropathy graded according to the modified Marsh scale [16], CD risk gradient based on HLA type [8], and HLA-DQ2 gene dose effect [7].

## Methods

### Study subjects and samples

Following written informed consent from parents/legal guardians, blood and duodenal biopsy specimens were collected from pediatric patients (Table 1) investigated for suspected CD or at follow-up on a gluten-free or gluten-containing diet, both for diagnostic purposes and for research purposes.

**Table 1:** **Descriptive data on study subjects including clinical antibody levels**

Group	No of cases ^a^(Females)	Age (years) median; 80% CR ^b^	Marsh grade span	Anti-TG2 (U/mL) ^c^median; 80% CR	Anti-DGP (U/mL) ^d^median; 80% CR	Anti-GL (U/mL) ^e^median; 80% CR
Not CD	14 (8)	8.8; 2.2-16	Marsh 0-1	0.25; 0.05-11	0.95; 0.20-20	0.60; 0.10-2.4
Normalized CD^f^	15 (13)	12; 7.3-17	Marsh 0-1	3.4; 0.40-28	2.6; 1.2-9.3	0.80; 0.20-4.1
Active CD^g^	20 (12)	5.9; 1.5-14	Marsh 2-3C	201; 4.4-1997	64; 5.6-392	16; 2.4-167
Under investigation^h^	3 (3)	15; 12-16	Marsh 0-1	15; 10-16	7.1; 2.1-22	1.9; 0.50-6.5

Study subjects are divided into groups depending on the diagnosis and the histopathologic assessment.

^a^Initially, a total of 56 cases were included in the study, but three cases were later excluded due to low sample amount and one case was excluded due to histopathologically non-assessable biopsies, leaving 52 cases included in the analyses.

^b^CR = Central range.

^c^Tissue transglutaminase autoantibodies (anti-TG2), positive result ≥ 7 U/mL.

^d^Deamidated gliadin antibodies (anti-DGP), positive result ≥ 10 U/mL.

^e^Gliadin antibodies (anti-GL), positive result ≥ 7 U/mL.

^f^Cases with confirmed celiac disease and duodenal biopsies indicating a Marsh grade 0–1, on a gluten-containing diet (n = 1) or a gluten-free diet (n = 14). Two cases had suboptimal (but assessable) biopsies.

^g^Cases with duodenal biopsies indicating a Marsh grade 2-3C, on a gluten-containing diet (n = 18) or a gluten-free diet (n = 2). One case had patchy lesions of the small intestine.

^h^Cases under continued gluten challenge and observation due to elevated antibody titers but normal mucosa. Ranges for this group are represented by minimum and maximum values.

For diagnostic purposes, blood was collected in a blood tube containing polymer gel and clot activator (Becton, Dickinson and Company, Franklin Lakes, NJ) and centrifuged at 2400 × g for 5 min for serum isolation. The sera were stored at room temperature for a maximum of 10 hours after centrifugation, and then at 4°C until the presence of CD-specific antibodies (anti-TG2 and antibodies against DGP [anti-DGP]) and antibodies against GL (anti-GL) was investigated, which occurred within 5 days of centrifugation. In six cases, the antibody tests were performed on plasma collected for research purposes. Multiple biopsy specimens were collected using an endoscope in all but one case, where a pediatric Watson capsule was used to extract a single biopsy specimen. The tissue was formalin-fixated and paraffin-embedded, and histopathologically assessed.

For research purposes, blood was collected both in EDTA blood tubes (Becton, Dickinson and Company) for DNA and plasma isolation, and in Tempus Blood RNA tubes (Life Technologies, Carlsbad, CA) for RNA isolation. An aliquot of EDTA blood for DNA isolation was removed, and the remaining blood centrifuged at 1500 × g for 10 min for plasma isolation. For two cases, one in the group with no indications of CD and one in the group with active CD (Table 1), blood for RNA purification was collected in EDTA blood tubes instead of Tempus tubes. Additionally, a biopsy specimen immersed in pre-chilled RNA*later* RNA Stabilization Reagent (Qiagen, Hilden, Germany) was collected from all cases in the study. Biopsies and stabilized blood for RNA purification were kept at 4°C for about 18 hours, and then at -20°C. RNA from EDTA blood was, however, purified without prior storage. Plasma was stored at -80°C. A maximum of two freeze-thaw cycles was accepted for all protein analyses.

The study was conducted under the approval of the Regional Ethical Review Board in Linköping.

### DNA purification

DNA was isolated from EDTA blood using the EZ1 DNA Blood 350 μL Kit and BioRobot EZ1 (Qiagen) according to the manufacturer’s instructions.

### RNA purification and reverse transcription

RNA from stabilized blood was purified using the Tempus Spin RNA Isolation Reagent kit (Life Technologies), and RNA from EDTA blood was purified using the QIAamp RNA Blood Mini kit (Qiagen), in both cases according to the manufacturer’s instructions. The quality of the RNA from stabilized blood and EDTA blood was verified, and the RNA was reverse transcribed using a previously documented procedure [15]. The resulting cDNA and the remaining RNA were stored at -80°C.

### Histopathologic assessment

Biopsies were assessed by a single experienced pathologist, blinded to all case data, in accordance with instructions for quality assurance and standardization assembled by the Swedish Society of Pathology. The status of the villi and crypts and the number of IELs were assessed for each biopsy. In cases where hematoxylin-eosin staining revealed an IEL number close to the ULN (25 IELs per 100 epithelial cells), an additional staining for CD3 was performed to better assess the number of IELs; when using CD3 staining, there should be >30 IELs per 100 epithelial cells to be indicative of CD. Hematoxylin-eosin staining was performed using the Tissue-Tek DRS 2000 Slide Stainer (Sakura, Alphen aan den Rijn, The Netherlands), and CD3 staining was performed using antibodies against CD3 (Dako, Glostrup, Denmark) and intelliPATH FLX (Biocare Medical, Concord, CA). The histological changes were reported according to the modified Marsh scale (0, 1, 2, 3A, 3B, or 3C) [16].

### Clinical antibody tests

Detection of IgA anti-TG2, IgA anti-GL, and Immunoglobulin G (IgG) anti-DGP in serum or plasma was performed using EliA Celikey IgA (positive result ≥ 7 U/mL), EliA Gliadin IgA (positive result ≥ 7 U/mL), and EliA GliadinDP IgG (positive result ≥ 10 U/mL), respectively, on Phadia250 (Thermo Fisher Scientific, Waltham, MA) as described by the manufacturer. In cases with total IgA levels below 0.07 g/L, detection of IgG anti-TG2 replaced IgA anti-TG2 (EliA Celikey IgG, Thermo Fisher Scientific). In order to distinguish results below the detection limit of an assay from missing data, the former were replaced with the detection limit divided by two.

### HLA typing and risk assessment

DNA from each case was HLA-typed for *DRB1*, *DQA1* and *DQB1* using a sequence-specific primer PCR method and capillary gel electrophoresis [17, 18]. The risk gradient for CD based on HLA type was calculated for each case using relative genotype risks extracted from a Scandinavian population [8].

### Selection of genes for analysis

Potential reference genes for the mRNA analysis were investigated using a Human Endogenous Control Plate (Life Technologies) containing assays for 32 potential reference genes, and cDNA from a total of nine blood RNA samples including three samples from cases with no mucosal injury (Marsh 0) and six with varying degrees of mucosal injury (Marsh 2-3C). Three potential reference genes (Additional file 1) were selected based on low sample-to-sample variation in mRNA levels, as investigated using the NormFinder algorithm [19] in version 5.4.2 of the Genex software package (MultiD Analyses, Göteborg, Sweden). The three selected reference genes were analyzed in the complete dataset, and the final selection of reference gene/s was established using the complete dataset by means of both low sample-to-sample variation and an absence of group differences in expression.

Genes for analysis of mRNA and protein levels were selected by reviewing published studies on blood mRNA/protein expression in CD, and by functional context (Additional file 1).

### Protein analysis

Multiplex detection of proteins in plasma was performed using Milliplex kits (Millipore, Billerica, MA) based on the Luminex xMAP technology, according to the manufacturer’s instructions (Additional file 1). The analyses were performed on the Bio-Plex 200 system (Bio-Rad, Hercules, CA). The *CD163* soluble protein was detected using an enzyme-linked immunosorbent assay (ELISA) according to instructions from the manufacturer (Additional file 1), and the results were obtained and analyzed using a Sunrise microplate absorbance reader combined with version 7.0 of the Magellan software package (Tecan Group Ltd, Männedorf, Switzerland). Standard curves were included in all protein analyses, and optimized using Bio-Plex Manager 6.1 (Bio-Rad) for all Milliplex assays, and the Akima method for curve fitting in Magellan v.7.0 for the ELISA. In order to distinguish results below the detection limit of an assay from missing data, the former were replaced with the lowest detected value of the assay divided by two.

### mRNA analysis

Levels of mRNA were investigated using custom made TaqMan Array Cards (Life Technologies) containing 47 gene expression assays including assays for the reference genes, or by using single assays and a previously documented procedure [15] (Additional file 1). Gene expression analysis on the TaqMan Array Cards was performed using the TaqMan Universal Master mix II without UNG and the recommended thermal profile (Life Technologies) on 300 ng cDNA in duplicates. Cards were prepared as recommended by the manufacturer, including analysis on the 7900HT Fast Real-time PCR system (Life Technologies).

For both TaqMan Array Cards and single assay results, quantification cycle (C_q_) values were established using version 1.0.2 of the ExpressionSuite software package (Life Technologies). The auto-baseline algorithm in the software was used to compensate for background noise for each amplification curve, and the thresholds were adjusted to the log-linear range and set to the same level for all samples within one assay. Missing C_q_ values due to low copy numbers were replaced by the highest C_q_ value available for the gene in question, increased by one cycle. The resulting C_q_ values were normalized against selected reference gene/s (Genex).

### Statistical analysis

Version 10 of the STATISTICA software package (StatSoft, Tulsa, OK) was used in all statistical analyses. Differential expression was investigated using Kruskal–Wallis one-way analysis of variance by ranks, except for the analysis of differential expression in cases stratified based on having one or two DQB1*02 alleles, where the Mann–Whitney U test was used. Post-hoc comparisons of mean ranks of all pairs of groups were performed (significance level p < 0.05, two-sided significance levels with a Bonferroni adjustment) [20]. Spearman rank correlation was used to investigate relations between mRNA/protein levels and histopathology and CD risk gradient based on HLA type. For all statistical analyses, except the post-hoc comparisons, a false-discovery rate was used and set to 5% [21].

The diagnostic performance of individual assays and logistic regression models of assay combinations was evaluated using receiver operating characteristic (ROC) curve analysis (MedCalc Statistical Software version 13.1.2, MedCalc Software, Ostend, Belgium).

## Results

### Differential blood marker expression

Detectable levels were found for all protein markers (n = 22, Additional file 1), and all mRNA markers except for *IL25* (n = 48, Additional file 1). *CDKN1B* was selected as the most stable reference gene, and used for normalization of all target mRNA levels.

**Figure 1:**
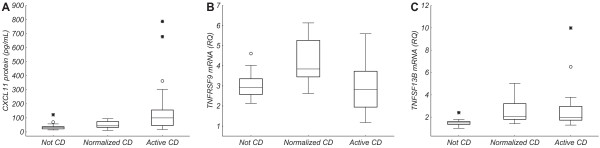
**Protein/messenger RNA (mRNA) levels of potential celiac disease (CD) markers.** Levels of **(A)** CXCL11 protein, **(B)** TNFRSF9 mRNA, and **(C)** TNFSF13B mRNA in cases without a CD diagnosis (Not CD), cases with confirmed CD and normalized duodenal histology (Normalized CD), and cases with active CD (Active CD). The box and the line represent the 25-75% interquartile range and the median, respectively. The whiskers represent the non-outlier range, open circles represent outliers, and stars represent extreme values.

In cases with active CD (Table 1, Active CD), significantly increased levels were observed for *CXCL11* protein (p = 0.003, Figure 1, Additional file 2) and *TNFSF13B* mRNA (p = 0.001, Figure 1, Additional file 2), in comparison to cases without a CD diagnosis (Table 1; Not CD). Additionally, *TNFSF13B* mRNA levels were significantly elevated in cases with confirmed CD and normalized histology (Table 1; Normalized CD), in comparison to cases without a CD diagnosis (p = 0.001, Figure 1, Additional file 2). Levels of *TNFRSF9* mRNA were significantly decreased in cases with active CD and in cases without a CD diagnosis, in comparison to CD cases with a normalized histology (p = 0.005 and p = 0.02, respectively, Figure 1, Additional file 2).

Previously established CD markers (anti-TG2, anti-DGP, and anti-GL) were differentially expressed in active CD cases in comparison to cases with normalized CD (p < 0.005) as well as cases without a CD diagnosis (p < 0.00005).

Stratification based on having one or two DQB1*02 alleles revealed no significant differences in levels of potential CD markers or in levels of previously established CD markers (anti-TG2, anti-DGP, and anti-GL), either when examining active CD cases only, or when looking at all CD cases together. Differences in expression in normalized CD cases alone could not be investigated, because all but two cases had one DQB1*02 allele.

### Correlation analysis

**Figure 2:**
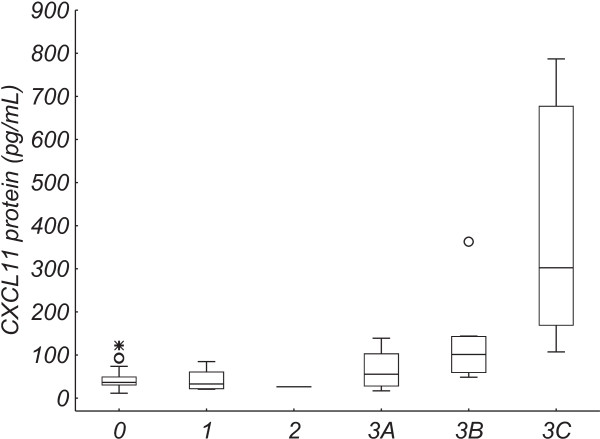
***CXCL11*** **protein levels** ***vs.*** **Marsh grade.** *CXCL11* protein levels for Marsh grades 0 (n = 25), 1 (n = 7), 2 (n = 1), 3A (n = 8), 3B (n = 6), and 3C (n = 5). The box and the line represent the 25-75% interquartile range and the median, respectively. The whiskers represent the non-outlier range, open circles represent outliers, and stars represent extreme values.

Significant correlations were observed between Marsh grade (all cases) and levels of C*XCL11* protein (Figure 2; Spearman rank correlation coefficient [r_s_] = 0.50), and *IL21* and *IL15* mRNA *(*r_s_ = -0.46 for both, data not shown).

**Figure 3:**
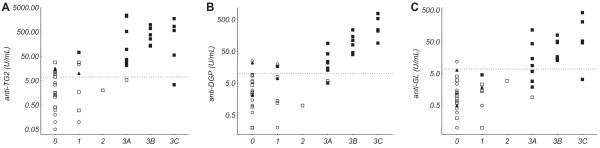
**Antibody levels** ***vs.*** **Marsh grade.** Antibody/autoantibody levels in serum/plasma against **A)** tissue transglutaminase (anti-TG2), **B)** deamidated gliadin (anti-DGP), and **C)** gliadin (anti-GL) *vs.* Marsh grade (0-3C) on a logarithmic scale. Open circles represent cases without a celiac disease (CD) diagnosis, open squares represent CD cases on a gluten-free diet, filled squares represent CD cases on a gluten-containing diet, and filled triangles represent cases under investigation for suspected CD. The dotted line represents the cut-off threshold for a positive result indicative of CD.

Clinical antibody levels (anti-TG2, anti-DGP, and anti-GL) displayed significant correlations with Marsh grade (r_s_ = 0.73, 0.75, and 0.75, respectively; Figure 3).

Levels of potential and previously established CD markers were not significantly correlated with HLA genotype risk for CD.

### Clinical antibody tests

All cases on a gluten-containing diet with anti-TG2 levels 10 times ULN or more (n = 13) showed a Marsh grade of 3A-3C and received a CD diagnosis. Correspondingly high levels of anti-DGP (n = 7) and anti-GL (n = 4) were less common, but all occurred in cases with an active CD diagnosis and a Marsh grade 3A-3C.

Of all cases with active CD on a gluten-containing diet (n = 18) and all cases without CD diagnosis (n = 14), anti-TG2 failed to identify one case with active CD (Marsh 3C, anti-TG2 3.4 U/mL) and gave a false positive result in two cases without CD diagnosis (Marsh 0–1, anti-TG2 11–23 U/mL).

Anti-DGP yielded the same number of misclassifications as anti-TG2, but affected partially different cases, whereas anti-GL yielded substantially more misclassifications with four false negative results, and one false positive result (data not shown).

Two cases with remaining enteropathy on a GFD (Marsh 2 - 3A, anti-TG2 2.0 - 5.4 U/mL) had normalized levels of all antibodies.

### Misclassified cases and cases under clinical investigation

The case with active CD on a gluten-containing diet that was misclassified based on levels of anti-TG2 (see Clinical antibody tests) showed levels of the two CD up-regulated markers *CXCL11* protein and *TNFSF13B* mRNA (Figure 1) that were above the 80% central range (CR) of the group without CD and within the 80% CR for the group with active CD (Additional file 2). For one of the two cases without CD that were misclassified based on levels of anti-TG2 (see Clinical antibody tests), levels of *CXCL11* protein were below the 80% CR of the group with active CD and thus corresponded to the “Not CD” group (Additional file 2).

One of the two CD cases with remaining enteropathy on a GFD that were misclassified based on anti-TG2 (see Clinical antibody tests) showed a level of the *TNFRSF9* mRNA marker for normalized CD (Figure 1) that was below the 80% CR of the group with normalized CD and within the 80% CR for the group with active CD (Additional file 2).

The remaining results fell within the 80% CR for more than one group.

Cases included in the group under investigation (Table 1, Under investigation) were on a gluten-containing diet and under continuous monitoring for suspected CD. Considering markers *CXCL11* protein and *TNFSF13B* mRNA, the median of the group fell within the 80% CR for both the group without a CD diagnosis and the group with active CD (Additional file 2).

### ROC curve analysis

**Figure 4:**
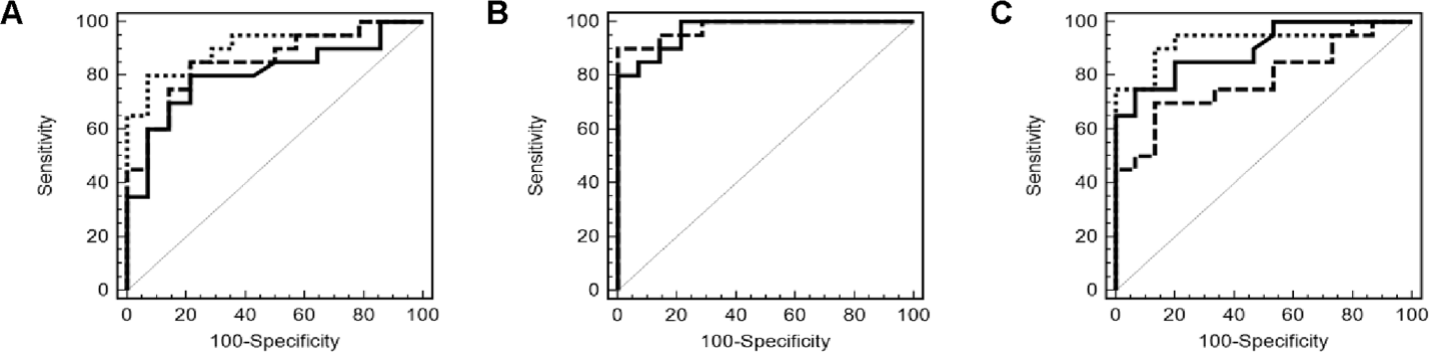
**Receiver operating characteristic (ROC) curves.** ROC curve analysis of discrimination between cases with active celiac disease (CD) and without CD **(A and B)** or between cases with active CD and normalized CD **(C)**. ROC curves in **(A)** correspond to *CXCL11* protein (solid line), *TNFSF13B* messenger RNA (mRNA) (dashed line), and a logistic regression model of *CXCL11* protein and *TNFSF13B* mRNA (dotted line). ROC curves in **(B)** correspond to tissue transglutaminase autoantibodies (anti-TG2) (solid line) and a logistic regression model of anti-TG2, *CXCL11* protein, and *TNFSF13B* mRNA (dashed line). ROC curves in **(C)** correspond to anti-TG2 (solid line), *TNFRSF9* mRNA (dashed line), and a logistic regression model of anti-TG2 and *TNFRSF9* mRNA (dotted line).

ROC curve analysis of discrimination between cases with active CD and without CD (Figure 4A and B) revealed a larger area under the curve (AUC) for anti-TG2 (AUC = 0.97) in comparison to *CXCL11* protein (AUC = 0.81), *TNFSF13B* mRNA (AUC = 0.85), and a logistic regression model based on *CXCL11* protein and *TNFSF13B* mRNA (AUC = 0.91). A logistic regression model based on anti-TG2, *CXCL11* protein, and *TNFSF13B* mRNA resulted in the highest AUC (0.98). However, compared to anti-TG2 alone, this improvement was not significant (p = 0.54). ROC curve analysis of discrimination between cases with active CD and normalized CD (Figure 4C) revealed an AUC of 0.90 for anti-TG2 and 0.78 for *TNFRSF9* mRNA. Compared to anti-TG2 alone, a logistic regression model based on anti-TG2 and *TNFRSF9* mRNA resulted in an increased AUC of 0.93 (p = 0.54).

## Discussion

The prospect of a blood-based diagnostic procedure for CD is appealing. This is reflected by the new recommendations from ESPGHAN, which offer the option to omit biopsies in patients with anti-TG2 titers of 10 times ULN or more [14]. In patients with high pretest probability for CD in combination with anti-TG2 levels of 10 times ULN or more, the probability of having CD is high, but it decreases somewhat in patients with lower pretest probabilities [22]. All patients with anti-TG2 levels of 10 times ULN or more in the current study were diagnosed with CD. However, in patients with lower antibody levels and/or of young age (<18 months), blood-based diagnostics seem to be less clear-cut [23, 24], and additional blood-based CD markers could be useful. Three potential blood-based CD markers (*CXCL11* protein, *TNFRSF9* mRNA, and *TNFSF13B* mRNA) were identified in the current study.

*CXCL11* protein, which is an IFN-γ and IFN-β induced chemokine [25], and mRNA from *TNFSF13B*, which encodes a cytokine with a major role in B cell growth and survival [26], showed elevated levels in cases with active CD compared to cases without a CD diagnosis, which is consistent with previous studies of *CXCL11* mRNA in small intestinal biopsies [15, 27] and *TNFSF13B* in serum [28]. An increased level of *TNFSF13B* mRNA was also evident in cases with CD and normalized histology compared to cases without a CD diagnosis, which is also consistent with previous results [28]. Increased serum levels of *TNFSF13B* protein have previously been found in autoimmune diseases [26], and *CXCL11* has also been implicated in autoimmune diseases [29].

*CXCL11* protein level correlated significantly with Marsh grade, as did mRNA levels of *IL21*, which is involved in the control of the innate and adaptive immune responses [30], and *IL15*, which is involved in the innate immune response in CD [31].

A decreased level of mRNA from *TNFRSF9*, which is a receptor induced on the surface of CD4+ and CD8+ T cells during activation [32], was found in cases with active CD and in cases without a CD diagnosis, compared with CD cases with normalized histology. This result differs from that in small intestinal biopsies [15], indicating tissue-specific regulation.

Potentially, *CXCL11* protein, *TNFRSF9* mRNA, and *TNFSF13B* mRNA might assist in the clinical diagnosis of CD. The established blood-based CD marker anti-TG2 misclassified one case with active CD and two cases without enteropathy on a gluten-containing diet, and two cases with remaining enteropathy on a GFD. In cases on a gluten-containing diet, *CXCL11* protein showed the greatest potential as a marker, with results in accordance with histopathology for two out of three cases misclassified by anti-TG2. For cases on a GFD with remaining enteropathy, results for *TNFRSF9* mRNA were in accordance with histopathology for one out of two cases.

ROC curve analysis showed that, as a single test, the already established anti-TG2 assay outperformed the new potential blood-based CD markers. However, it might be possible to increase the diagnostic performance by considering several assay results jointly. Adding new markers to the anti-TG2 assay produced a slight, though non-significant, increase in diagnostic performance. At present, the data is too limited for any firm conclusions regarding added diagnostic value, and our analysis provided no suggestions as to the potential CD status of cases in the group under investigation.

Several proteins in serum/plasma have previously been suggested as potential blood-based CD markers, for example regenerating gene Iα protein and intestinal fatty acid binding protein [33, 34]. Recently, Galatola *et al.* proposed a discriminant model based on the expression of four genes including *REL* and *TNFAIP3* [35], which along with *TNFSF13B* and *TNFRSF9* (identified as potential CD markers in the current study) are involved in regulation of the NF-κB complex [32, 36, 37]. Furthermore, the NF-κB complex is involved in the IFN-β induced transcription of *CXCL11* [38], and *REL* is involved in the transcriptional activation of *TNFSF13B* [26]. This indicates that additional investigations into NF-κB interacting proteins could reveal new potential markers for diagnosis and monitoring of CD.

## Conclusions

The CD markers identified in this study further emphasize the significance of components related to NF-κB regulation in relation to CD. However, the diagnostic relevance of *CXCL11*, *TNFSF13B*, *TNFRSF9*, and other NF-κB interacting proteins recognized by pathway analysis needs to be further investigated.

### Human genes

The human genes discussed in this article are presented in Additional file 1.

## Electronic supplementary material

Additional file 1: **Genes selected for messenger RNA (mRNA) and/or protein detection in human blood.** Protein/mRNA levels were investigated in cases with active celiac disease (CD), cases with confirmed CD and normalized histology, cases without a CD diagnosis, and cases under investigation for suspected CD. Some genes were selected based on their context; others were selected based on information from published studies. (DOCX 34 KB)

Additional file 2: **Descriptive data on differentially expressed potential blood-based celiac disease (CD) markers.** Study subjects are divided into groups depending on the diagnosis and the histopathologic assessment. (DOCX 18 KB)

## Abbreviations

CD: Celiac disease
TG2: Tissue transglutaminase
DGP: Deamidated gliadin
GL: Native gliadin
IELs: Intraepithelial lymphocytes
anti-TG2: Autoantibodies against tissue transglutaminase
ESPGHAN: European Society of Paediatric Gastroenterology, Hepatology and Nutrition
ULN: Upper limit of normal
mRNA: Messenger RNA
anti-DGP: Antibodies against deamidated gliadin
anti-GL: Antibodies against native gliadin
C_q_: Quantification cycle
ROC: Receiver operating characteristic
CR: Central range
AUC: Area under the curve.

## References

[1] Ludvigsson JF, Leffler DA, Bai JC, Biagi F, Fasano A, Green PH, Hadjivassiliou M, Kaukinen K, Kelly CP, Leonard JN, Lundin KE, Murray JA, Sanders DS, Walker MM, Zingone F, Ciacci C (2013). The Oslo definitions for coeliac disease and related terms. Gut.

[2] Tack GJ, Verbeek WH, Schreurs MW, Mulder CJ (2010). The spectrum of celiac disease: epidemiology, clinical aspects and treatment. Nat Rev Gastroenterol Hepatol.

[3] van Heel DA, Franke L, Hunt KA, Gwilliam R, Zhernakova A, Inouye M, Wapenaar MC, Barnardo MC, Bethel G, Holmes GK, Feighery C, Jewell D, Kelleher D, Kumar P, Travis S, Walters JR, Sanders DS, Howdle P, Swift J, Playford RJ, McLaren WM, Mearin ML, Mulder CJ, McManus R, McGinnis R, Cardon LR, Deloukas P, Wijmenga C (2007). A genome-wide association study for celiac disease identifies risk variants in the region harboring IL2 and IL21. Nat Genet.

[4] Dubois PC, Trynka G, Franke L, Hunt KA, Romanos J, Curtotti A, Zhernakova A, Heap GA, Adany R, Aromaa A, Bardella MT, van den Berg LH, Bockett NA, de la Concha EG, Dema B, Fehrmann RS, Fernandez-Arquero M, Fiatal S, Grandone E, Green PM, Groen HJ, Gwilliam R, Houwen RH, Hunt SE, Kaukinen K, Kelleher D, Korponay-Szabo I, Kurppa K, MacMathuna P, Maki M (2010). Multiple common variants for celiac disease influencing immune gene expression. Nat Genet.

[5] Sollid LM, Markussen G, Ek J, Gjerde H, Vartdal F, Thorsby E (1989). Evidence for a primary association of celiac disease to a particular HLA-DQ alpha/beta heterodimer. J Exp Med.

[6] Sollid LM (2002). Coeliac disease: dissecting a complex inflammatory disorder. Nat Rev Immunol.

[7] Ploski R, Ek J, Thorsby E, Sollid LM (1993). On the HLA-DQ(alpha 1*0501, beta 1*0201)-associated susceptibility in celiac disease: a possible gene dosage effect of DQB1*0201. Tissue Antigens.

[8] Margaritte-Jeannin P, Babron MC, Bourgey M, Louka AS, Clot F, Percopo S, Coto I, Hugot JP, Ascher H, Sollid LM, Greco L, Clerget-Darpoux F (2004). HLA-DQ relative risks for coeliac disease in European populations: a study of the European genetics cluster on celiac disease. Tissue Antigens.

[9] Megiorni F, Mora B, Bonamico M, Barbato M, Nenna R, Maiella G, Lulli P, Mazzilli MC (2009). HLA-DQ and risk gradient for celiac disease. Hum Immunol.

[10] Hunt KA, Zhernakova A, Turner G, Heap GA, Franke L, Bruinenberg M, Romanos J, Dinesen LC, Ryan AW, Panesar D, Gwilliam R, Takeuchi F, McLaren WM, Holmes GK, Howdle PD, Walters JR, Sanders DS, Playford RJ, Trynka G, Mulder CJ, Mearin ML, Verbeek WH, Trimble V, Stevens FM, O’Morain C, Kennedy NP, Kelleher D, Pennington DJ, Strachan DP, McArdle WL (2008). Newly identified genetic risk variants for celiac disease related to the immune response. Nat Genet.

[11] Trynka G, Hunt KA, Bockett NA, Romanos J, Mistry V, Szperl A, Bakker SF, Bardella MT, Bhaw-Rosun L, Castillejo G, de la Concha EG, de Almeida RC, Dias KR, van Diemen CC, Dubois PC, Duerr RH, Edkins S, Franke L, Fransen K, Gutierrez J, Heap GA, Hrdlickova B, Hunt S, Plaza Izurieta L, Izzo V, Joosten LA, Langford C, Mazzilli MC, Mein CA, Midah V (2011). Dense genotyping identifies and localizes multiple common and rare variant association signals in celiac disease. Nat Genet.

[12] Ostensson M, Monten C, Bacelis J, Gudjonsdottir AH, Adamovic S, Ek J, Ascher H, Pollak E, Arnell H, Browaldh L, Agardh D, Wahlstrom J, Nilsson S, Torinsson-Naluai A (2013). A possible mechanism behind autoimmune disorders discovered by genome-wide linkage and association analysis in celiac disease. PLoS One.

[13] Kneepkens CM, von Blomberg BM (2012). Clinical practice: celiac disease. Eur J Pediatr.

[14] Husby S, Koletzko S, Korponay-Szabo IR, Mearin ML, Phillips A, Shamir R, Troncone R, Giersiepen K, Branski D, Catassi C, Lelgeman M, Maki M, Ribes-Koninckx C, Ventura A, Zimmer KP (2012). European Society for Pediatric Gastroenterology, Hepatology, and Nutrition guidelines for the diagnosis of celiac disease. J Pediatr Gastroenterol Nutr.

[15] Bragde H, Jansson U, Jarlsfelt I, Soderman J (2011). Gene expression profiling of duodenal biopsies discriminates celiac disease mucosa from normal mucosa. Pediatr Res.

[16] Rostami K, Kerckhaert J, Tiemessen R, von Blomberg BM, Meijer JW, Mulder CJ (1999). Sensitivity of antiendomysium and antigliadin antibodies in untreated celiac disease: disappointing in clinical practice. Am J Gastroenterol.

[17] Lavant EH, Carlson JA (2009). A new automated human leukocyte antigen genotyping strategy to identify DR-DQ risk alleles for celiac disease and type 1 diabetes mellitus. Clin Chem Lab Med.

[18] Lavant EH, Agardh DJ, Nilsson A, Carlson JA (2011). A new PCR-SSP method for HLA DR-DQ risk assessment for celiac disease. Clin Chim Acta.

[19] Andersen CL, Jensen JL, Orntoft TF (2004). Normalization of real-time quantitative reverse transcription-PCR data: a model-based variance estimation approach to identify genes suited for normalization, applied to bladder and colon cancer data sets. Cancer Res.

[20] Siegel S, Castellan NJ (1988). Nonparametric statistics for the behavioral sciences.

[21] Benjamini Y, Hochberg Y (1995). Controlling the false discovery rate: a practical and powerful approach to multiple testing. J Roy Stat Soc B.

[22] Vermeersch P, Geboes K, Marien G, Hoffman I, Hiele M, Bossuyt X (2013). Defining thresholds of antibody levels improves diagnosis of celiac disease. Clin Gastroenterol Hepatol.

[23] Giersiepen K, Lelgemann M, Stuhldreher N, Ronfani L, Husby S, Koletzko S, Korponay-Szabo IR (2012). Accuracy of diagnostic antibody tests for coeliac disease in children: summary of an evidence report. J Pediatr Gastroenterol Nutr.

[24] Lagerqvist C, Dahlbom I, Hansson T, Jidell E, Juto P, Olcen P, Stenlund H, Hernell O, Ivarsson A (2008). Antigliadin immunoglobulin A best in finding celiac disease in children younger than 18 months of age. J Pediatr Gastroenterol Nutr.

[25] Rani MR, Foster GR, Leung S, Leaman D, Stark GR, Ransohoff RM (1996). Characterization of beta-R1, a gene that is selectively induced by interferon beta (IFN-beta) compared with IFN-alpha. J Biol Chem.

[26] Lahiri A, Pochard P, Le Pottier L, Tobon GJ, Bendaoud B, Youinou P, Pers JO (2012). The complexity of the BAFF TNF-family members: implications for autoimmunity. J Autoimmun.

[27] Diosdado B, van Bakel H, Strengman E, Franke L, van Oort E, Mulder CJ, Wijmenga C, Wapenaar MC (2007). Neutrophil recruitment and barrier impairment in celiac disease: a genomic study. Clin Gastroenterol Hepatol.

[28] Fabris M, Visentini D, De Re V, Picierno A, Maieron R, Cannizzaro R, Villalta D, Curcio F, De Vita S, Tonutti E (2007). Elevated B cell-activating factor of the tumour necrosis factor family in coeliac disease. Scand J Gastroenterol.

[29] Lacotte S, Brun S, Muller S, Dumortier H (2009). CXCR3, inflammation, and autoimmune diseases. Ann N Y Acad Sci.

[30] Sarra M, Cupi ML, Pallone F, Monteleone G (2012). Interleukin-21 in immune and allergic diseases. Inflamm Allergy Drug Targets.

[31] De Nitto D, Monteleone I, Franze E, Pallone F, Monteleone G (2009). Involvement of interleukin-15 and interleukin-21, two gamma-chain-related cytokines, in celiac disease. World J Gastroenterol.

[32] Jang IK, Lee ZH, Kim YJ, Kim SH, Kwon BS (1998). Human 4-1BB (CD137) signals are mediated by TRAF2 and activate nuclear factor-kappa B. Biochem Biophys Res Commun.

[33] Adriaanse MP, Tack GJ, Passos VL, Damoiseaux JG, Schreurs MW, van Wijck K, Riedl RG, Masclee AA, Buurman WA, Mulder CJ, Vreugdenhil AC (2013). Serum I-FABP as marker for enterocyte damage in coeliac disease and its relation to villous atrophy and circulating autoantibodies. Aliment Pharmacol Ther.

[34] Planas R, Pujol-Autonell I, Ruiz E, Montraveta M, Cabre E, Lucas-Martin A, Pujol-Borrell R, Martinez-Caceres E, Vives-Pi M (2011). Regenerating gene Ialpha is a biomarker for diagnosis and monitoring of celiac disease: a preliminary study. Transl Res.

[35] Galatola M, Izzo V, Cielo D, Morelli M, Gambino G, Zanzi D, Strisciuglio C, Sperandeo MP, Greco L, Auricchio R (2013). Gene expression profile of peripheral blood monocytes: a step towards the molecular diagnosis of celiac disease?. PLoS One.

[36] Rickert RC, Jellusova J, Miletic AV (2011). Signaling by the tumor necrosis factor receptor superfamily in B-cell biology and disease. Immunol Rev.

[37] Trynka G, Zhernakova A, Romanos J, Franke L, Hunt KA, Turner G, Bruinenberg M, Heap GA, Platteel M, Ryan AW, de Kovel C, Holmes GK, Howdle PD, Walters JR, Sanders DS, Mulder CJ, Mearin ML, Verbeek WH, Trimble V, Stevens FM, Kelleher D, Barisani D, Bardella MT, McManus R, van Heel DA, Wijmenga C (2009). Coeliac disease-associated risk variants in TNFAIP3 and REL implicate altered NF-kappaB signalling. Gut.

[38] Rani MR, Asthagiri AR, Singh A, Sizemore N, Sathe SS, Li X, DiDonato JD, Stark GR, Ransohoff RM (2001). A role for NF-kappa B in the induction of beta-R1 by interferon-beta. J Biol Chem.

[CR39] The pre-publication history for this paper can be accessed here:http://www.biomedcentral.com/1471-230X/14/176/prepub

