# Supplementary material for: Potential blood-based markers of celiac disease
Source: BMC Gastroenterol. 2014 Oct 9;14:176. doi: 10.1186/1471-230X-14-176 (PMC4287385; doi:10.1186/1471-230X-14-176)
Supplement: Supplementary file 1 — Additional file 1: Genes selected for messenger RNA (mRNA) and/or protein detection in human blood. Protein/mRNA levels were investigated in cases with active celiac disease (CD), cases with confirmed CD and normalized histology, cases without a CD diagnosis, and cases under investigation for suspected CD. Some genes were selected based on their context; others were selected based on information from published studies. (DOCX 34 KB) [file 12876_2014_1197_MOESM1_ESM.docx]

| Gene symbol | Gene name | mRNA assay^a^ | Reference sequence/s | mRNA Assay location^b^ | Protein assay^c^ | Protein name |
| --- | --- | --- | --- | --- | --- | --- |
| ***Reference genes*** | | | | | | |
| *CDKN1B* | cyclin-dependent kinase inhibitor 1B (p27, Kip1) | Hs00153277_m1 | NM_004064.3 | 950 |  |  |
| *PUM1* | pumilio homolog 1 | Hs00206469_m1 | NM_001020658.1 | 106 |  |  |
| *RPS17* | ribosomal protein S17 | Hs00734303_g1 | NM_001021.3 | 356 |  |  |
| ***Candidate marker genes*** | | | | | | |
| *ADM [1]* | adrenomedullin | Hs00181605_m1 | NM_001124.1 | 136 |  |  |
| *APOA1 [2]* | apolipoprotein A-I |  |  |  | Multiplex | apolipoprotein A-I |
| *APOB [3]* | apolipoprotein B |  |  |  | Multiplex | apolipoprotein B-100 |
| *APOC3* | apolipoprotein C-III |  |  |  | Multiplex | apolipoprotein C-III |
| *AQP9 [1]* | aquaporin 9 | Hs01035888_m1 | NM_020980.3 | 1065 |  |  |
| *BCL6 [4]* | B-cell CLL/lymphoma 6 | Hs00277037_m1 | NM_001130845.1 | 2434 |  |  |
|  |  |  | NM_001134738.1 | 1858 |  |  |
|  |  |  | NM_001706.4 | 2342 |  |  |
| *CCL2 [5]* | chemokine (C-C motif) ligand 2 | Hs00234140_m1 | NM_002982.3 | 151 |  |  |
| *CCL3** | chemokine (C-C motif) ligand 3 | Hs00234142_m1 | NM_002983.2 | 175 | Multiplex | C-C motif chemokine 3 |
| *CCL4** | chemokine (C-C motif) ligand 4 | Hs01031494_m1 | AY312578.1 | 89 | Multiplex | C-C motif chemokine 4 |
|  |  |  | AY766448.1 | 157 |  |  |
| *CCL5 [6]* | chemokine (C-C motif) ligand 5 | Hs00174575_m1 | NM_002985.2 | 253 |  |  |
| *CCL20* [7]* | chemokine (C-C motif) ligand 20 | Hs01011368_m1 | NM_001130046.1 | 330 | Multiplex | C-C motif chemokine 20 |
|  |  |  | NM_004591.2 | 333 |  |  |
| *CCL25 [8]* | chemokine (C-C motif) ligand 25 | Hs00171144_m1 | NM_005624.2 | 445 |  |  |
| *CCR3* | chemokine (C-C motif) receptor 3 | Hs00266213_s1 | NM_178329.2 | 289 |  |  |
|  |  |  | NM_178328.1 | 358 |  |  |
|  |  |  | NM_001164680.1 | 349 |  |  |
|  |  |  | NM_001837.3 | 368 |  |  |
| *CCR5 [9]* | chemokine (C-C motif) receptor 5 | Hs00152917_m1 | NM_001100168.1 | 110 |  |  |
|  |  |  | NM_000579.3 | 345 |  |  |
| *CCR6 [7]* | chemokine (C-C motif) receptor 6 | Hs01890706_s1 | NM_031409.3 | 2774 |  |  |
|  |  |  | NM_004367.5 | 2511 |  |  |
| *CCR9 [8]* | chemokine (C-C motif) receptor 9 | Hs01890924_s1 | NM_031200.2 | 2397 |  |  |
|  |  |  | NR_036525.1 | 2483 |  |  |
|  |  |  | NM_006641.3 | 2348 |  |  |
| *CD163 [10]* | CD163 molecule | Hs00174705_m1 | NM_203416.2 | 1547 | ELISA | scavenger receptor cysteine-rich type 1 protein M130 |
|  |  |  | NM_004244.4 | 1547 |  |  |
| *CD1d [11]* | CD1d molecule | Hs00939888_m1 | NM_001766.3 | 1381 |  |  |
| *CXCL10* | chemokine (C-X-C motif) ligand 10 | Hs00171042_m1 | NM_001565.2 | 133 | Multiplex | C-X-C motif chemokine 10 |
| *CXCL11* | chemokine (C-X-C motif) ligand 11 | Hs00171138_m1 | NM_005409.4 | 269 | Multiplex | C-X-C motif chemokine 11 |
| *CXCL2 [1]* | chemokine (C-X-C motif) ligand 2 | Hs00601975_m1 | NM_002089.3 | 473 |  |  |
| *FAS [12]* | Fas (TNF receptor superfamily, member 6) | Hs00537680_m1 | NM_152871.1 | 852 |  |  |
|  |  |  | NR_028033.1 | 743 |  |  |
|  |  |  | NR_028034.1 | 605 |  |  |
| *GZMB* [13]* | granzyme B (granzyme 2, cytotoxic T-lymphocyte-associated serine esterase 1) | Hs00188051_m1 | NM_004131.4 | 664 | Multiplex | granzyme B |
| *IFNG [14]* | interferon, gamma | Hs00174143_m1 | NM_000619.2 | 246 | Multiplex | interferon gamma |
| *IL2 [14]* | interleukin 2 | Hs00174114_m1 | NM_000586.3 | 267 | Multiplex | interleukin-2 |
| *IL2RA [15]* | Soluble IL-2Rα | Hs00907779_m1 | NM_000417.2 | 799 |  |  |
| *IL4 [14]* | Interleukin 4 | Hs99999030_m1 | NM_172348.1 | 684 | Multiplex | interleukin-4 |
|  |  |  | NM_000589.2 | 732 |  |  |
| *IL6 [14]* | interleukin 6 (interferon, beta 2) | Hs00985639_m1 | NM_000600.3 | 321 | Multiplex | interleukin-6 |
| *IL7* | interleukin 7 | Hs00174202_m1 | NM_001199886.1 | 752 | Multiplex | interleukin-7 |
|  |  |  | NM_001199887.1 | 752 |  |  |
|  |  |  | NM_001199888.1 | 752 |  |  |
|  |  |  | NM_000880.3 | 752 |  |  |
| *IL8 [14]* | interleukin 8 | Hs00174103_m1 | NM_000584.2 | 170 | Multiplex | interleukin-8 |
| *IL10 [14]* | interleukin 10 | Hs00174086_m1 | NM_000572.2 | 441 | Multiplex | interleukin-10 |
| *IL11* | interleukin 11 | Hs00174148_m1 | NM_000641.2 | 314 | Multiplex | interleukin-11 |
| *IL12B* | interleukin 12B (natural killer cell stimulatory factor 2, cytotoxic lymphocyte maturation factor 2, p40) | Hs01011516_g1 | NM_002187.2 | 399 | Multiplex | interleukin-12 subunit beta |
| *IL15* | interleukin 15 | Hs01003716_m1 | NM_172174.2 | 1224 | Multiplex | interleukin-15 |
|  |  |  | NM_000585.3 | 748 |  |  |
| *IL17A* | interleukin 17A | Hs00174383_m1 | NM_002190.2 | 279 | Multiplex | interleukin-17A |
| *IL17F [16]* | interleukin 17F | Hs00369400_m1 | NM_052872.3 | 323 |  |  |
| *IL18* | interleukin 18 | Hs00155517_m1 | NM_001562.2 | 584 |  |  |
| *IL21* | interleukin 21 | Hs00222327_m1 | NM_021803.2 | 405 |  |  |
| *IL23A* | interleukin 23, alpha subunit p19 | Hs00900828_g1 | NM_016584.2 | 429 |  |  |
| *IL25* | Interleukin 25 | Hs03044841_m1 | NM_172314.1 | 386 |  |  |
|  |  |  | NM_022789.3 | 534 |  |  |
| *ITGB7 [17]* | integrin, beta 7 | Hs01565750_m1 | NM_000889.1 | 1306 |  |  |
| *KRT19* | Keratin 19 | Hs01051611_gH | NM_002276.4 | 1090 |  |  |
| *MAPK1 [18]* | mitogen-activated protein kinase 1 | Hs01046830_m1 | NM_138957.2 | 1100 |  |  |
|  |  |  | NM_002745.4 | 1100 |  |  |
| *MMP8** | matrix metallopeptidase 8 (neutrophil collagenase) | Hs01029057_m1 | NM_002424.2 | 876 | Multiplex | neutrophil collagenase |
| *NAMPT [1]* | nicotinamide phosphoribosyltransferase | Hs00237184_m1 | NM_005746.2 | 360 |  |  |
| *OCLN* | Occludin | Hs00170162_m1 | NM_002538.2 | 1474 |  |  |
| *SELL [19]* | selectin L | Hs00174151_m1 | NM_000655.4 | 211 |  |  |
| *TFF3 [20]* | trefoil factor 3 (intestinal) | Hs00902278_m1 | NM_003226.3 | 451 |  |  |
| *TLR2 [19]* | toll-like receptor 2 | Hs00610101_m1 | NM_003264.3 | 198 |  |  |
| *TLR4 [19]* | toll-like receptor 4 | Hs01060206_m1 | NM_138554.3 | 381 |  |  |
| *TNFRSF9* | tumor necrosis factor receptor superfamily, member 9 | Hs00155512_m1 | NM_001561.5 | 675 |  |  |
| *TNFSF13B [21]* | tumor necrosis factor (ligand) superfamily, member 13b | Hs00902572_g1 | NM_001145645.1 | 607 |  |  |
|  |  |  | NM_006573.3 | 607 |  |  |

^a^Messenger RNA was detected using reverse transcription quantitative PCR and TaqMan Array Micro Fluidic Cards, or single TaqMan Gene Expression assays (single assays indicated with an asterisk) and total RNA (converted to complementary DNA) from human blood. Final concentrations of primers and probe were 900 nM and 250 nM, respectively. The probes are minor groove binding TaqMan probes, fluorescently labeled in the 5’ end with FAM and in the 3’ end with a non-fluorescent quencher.

^b^Midpoint of the 25 bp context sequence which contains the probe sequence.

^c^Proteins were detected using a multiplex format (Luminex xMAP technology) or an enzyme-linked immunosorbent assay (ELISA).

**References**

1. Mesko B, Poliska S, Szegedi A, Szekanecz Z, Palatka K, Papp M, Nagy L: **Peripheral blood gene expression patterns discriminate among chronic inflammatory diseases and healthy controls and identify novel targets**. *BMC Med Genomics* 2010, **3**:15.

2. Floren CH, Alm P: **Defective synthesis of apolipoprotein A-I in jejunal mucosa in coeliac disease**. *Scand J Gastroenterol* 1988, **23**(7):856-860.

3. Brandimarte G, Tursi A: **Regression of Apo B deficiency in biovular twins with Apo B deficiency and celiac disease after gluten withdrawal**. *Am J Gastroenterol* 2002, **97**(7):1856-1858.

4. Mondal A, Sawant D, Dent AL: **Transcriptional repressor BCL6 controls Th17 responses by controlling gene expression in both T cells and macrophages**. *J Immunol* 2010, **184**(8):4123-4132.

5. Yamashiro S, Kamohara H, Wang JM, Yang D, Gong WH, Yoshimura T: **Phenotypic and functional change of cytokine-activated neutrophils: inflammatory neutrophils are heterogeneous and enhance adaptive immune responses**. *J Leukoc Biol* 2001, **69**(5):698-704.

6. Palova-Jelinkova L, Rozkova D, Pecharova B, Bartova J, Sediva A, Tlaskalova-Hogenova H, Spisek R, Tuckova L: **Gliadin fragments induce phenotypic and functional maturation of human dendritic cells**. *J Immunol* 2005, **175**(10):7038-7045.

7. Fasano A, Troncone R, Branski D (eds.): **Frontiers in Celiac Disease**. Basel: Karger; 2008.

8. Papadakis KA, Landers C, Prehn J, Kouroumalis EA, Moreno ST, Gutierrez-Ramos JC, Hodge MR, Targan SR: **CC chemokine receptor 9 expression defines a subset of peripheral blood lymphocytes with mucosal T cell phenotype and Th1 or T-regulatory 1 cytokine profile**. *J Immunol* 2003, **171**(1):159-165.

9. Smyth DJ, Plagnol V, Walker NM, Cooper JD, Downes K, Yang JH, Howson JM, Stevens H, McManus R, Wijmenga C, Heap GA, Dubois PC, Clayton DG, Hunt KA, van Heel DA, Todd JA: **Shared and distinct genetic variants in type 1 diabetes and celiac disease**. *N Engl J Med* 2008, **359**(26):2767-2777.

10. Daly A, Walsh C, Feighery C, O'Shea U, Jackson J, Whelan A: **Serum levels of soluble CD163 correlate with the inflammatory process in coeliac disease**. *Aliment Pharmacol Ther* 2006, **24**(3):553-559.

11. Grose RH, Cummins AG, Thompson FM: **Deficiency of invariant natural killer T cells in coeliac disease**. *Gut* 2007, **56**(6):790-795.

12. Di Sabatino A, D'Alo S, Millimaggi D, Ciccocioppo R, Parroni R, Sciarra G, Cifone MG, Corazza GR: **Apoptosis and peripheral blood lymphocyte depletion in coeliac disease**. *Immunology* 2001, **103**(4):435-440.

13. Augustin MT, Kokkonen J, Karttunen R, Karttunen TJ: **Serum granzymes and CD30 are increased in children's milk protein sensitive enteropathy and celiac disease**. *J Allergy Clin Immunol* 2005, **115**(1):157-162.

14. Manavalan JS, Hernandez L, Shah JG, Konikkara J, Naiyer AJ, Roland Lee A, Ciaccio E, Minaya MT, Green PH, Bhagat G: **Serum cytokine elevations in celiac disease: Association with disease presentation**. *Hum Immunol* 2009.

15. Lahat N, Shapiro S, Karban A, Gerstein R, Kinarty A, Lerner A: **Cytokine profile in coeliac disease**. *Scand J Immunol* 1999, **49**(4):441-446.

16. Chang SH, Dong C: **IL-17F: regulation, signaling and function in inflammation**. *Cytokine* 2009, **46**(1):7-11.

17. Di Sabatino A, Rovedatti L, Rosado MM, Carsetti R, Corazza GR, MacDonald TT: **Increased expression of mucosal addressin cell adhesion molecule 1 in the duodenum of patients with active celiac disease is associated with depletion of integrin alpha4beta7-positive T cells in blood**. *Human pathology* 2009, **40**(5):699-704.

18. Broide E, Scapa E, Bloch O, Shapiro M, Kimchi NA, Ben-Yehudah G, Rapoport MJ: **Evidence for aberrant regulation of MAP kinase signal transduction pathway in peripheral blood mononuclear cells in patients with active celiac disease**. *Dig Dis Sci* 2009, **54**(6):1270-1275.

19. Cseh A, Vasarhelyi B, Szalay B, Molnar K, Nagy-Szakal D, Treszl A, Vannay A, Arato A, Tulassay T, Veres G: **Immune Phenotype of Children with Newly Diagnosed and Gluten-Free Diet-Treated Celiac Disease**. *Dig Dis Sci* 2010.

20. Ciacci C, Di Vizio D, Seth R, Insabato G, Mazzacca G, Podolsky DK, Mahida YR: **Selective reduction of intestinal trefoil factor in untreated coeliac disease**. *Clin Exp Immunol* 2002, **130**(3):526-531.

21. Fabris M, Visentini D, De Re V, Picierno A, Maieron R, Cannizzaro R, Villalta D, Curcio F, De Vita S, Tonutti E: **Elevated B cell-activating factor of the tumour necrosis factor family in coeliac disease**. *Scand J Gastroenterol* 2007:1-6.
