# Supplementary material for: Potential blood-based markers of celiac disease
Source: BMC Gastroenterol. 2014 Oct 9;14:176. doi: 10.1186/1471-230X-14-176 (PMC4287385; doi:10.1186/1471-230X-14-176)
Supplement: Supplementary file 2 — Additional file 2: Descriptive data on differentially expressed potential blood-based celiac disease (CD) markers. Study subjects are divided into groups depending on the diagnosis and the histopathologic assessment. (DOCX 18 KB) [file 12876_2014_1197_MOESM2_ESM.docx]

| Group | *CXCL11* protein (pg/mL) Median; 80% CR^a^ | *TNFRSF9* mRNA (RQ)^b^ Median; 80% CR | *TNFSF13B* mRNA (RQ) Median; 80% CR |
| --- | --- | --- | --- |
| Not CD | 32; 15-70 | 2.9; 2.2-4.0 | 1.5; 1.2-1.8 |
| Normalized CD | 43; 21-92 | 3.8; 2.9-5.9 | 2.0; 1.6-4.4 |
| Active CD | 99; 23-520 | 2.8; 1.4-4.7 | 2.0; 1.5-5.1 |
| Under investigation^c^ | 45; 33-50 | 3.6; 3.3-5.2 | 1.8; 1.5-2.2 |

^a^CR = Central range

^b^RQ = Relative quantity of messenger RNA (mRNA), normalized against reference gene *CDKN1B*.

^c^Ranges for this group are represented by minimum and maximum values.
